# Supplementary material for: The antiaging effects of a product containing collagen and ascorbic acid: In vitro, ex vivo, and pre-post intervention clinical trial
Source: PLoS One. 2022 Dec 12;17(12):e0277188. doi: 10.1371/journal.pone.0277188 (PMC9744321; doi:10.1371/journal.pone.0277188)
Supplement: S1 Table — (DOCX) [file pone.0277188.s002.docx]

**S1 Table. *p*-Values for the Graphs Included in the Figures.**

**For data in Fig 2D.** Analysis of collagen synthesis depending on the concentration of the test product.

| *p*-value | | |
| --- | --- | --- |
| 0.01% | 0.02% | 0.05% |
| 0.014 | 0.000 | 0.001 |

**For data in Fig 3.** Analysis of parameters after treatment for 4 weeks

3 A1. Crow’s feet

| Parameter | *p*-value | |
| --- | --- | --- |
|  | 2 weeks | 4 weeks |
| 1) Average depth of wrinkles | 0.001 | 0.000 |
| 2) Mean depth biggest wrinkle | 0.000 | 0.000 |
| 3) Max. depth biggest wrinkle | 0.000 | 0.000 |
| 4) Total wrinkle count | 0.029 | 0.022 |
| 5) Total wrinkle volume | 0.000 | 0.000 |
| 6) Total wrinkle area | 0.049 | 0.035 |
| 7) Total length of wrinkles | 0.003 | 0.027 |
| 8) Ra | 0.000 | 0.000 |
| 9) Rz | 0.001 | 0.000 |

3 A2. Nasolabial fold and forehead

| Parameter | *p*-value | |
| --- | --- | --- |
|  | 2 weeks | 4 weeks |
| 1) Average depth of wrinkles | 0.001 | 0.0000 |
| 2) Mean depth biggest wrinkle | 0.002 | 0.000 |
| 3) Max. depth biggest wrinkle | 0.001 | 0.000 |
| 4) Total wrinkle count | 0.008 | 0.000 |
| 5) Total wrinkle volume | 0.012 | 0.002 |
| 6) Total wrinkle area | 0.018 | 0.029 |
| 7) Total length of wrinkles | 0.003 | 0.001 |
| 8) Ra | 0.002 | 0.000 |
| 9) Rz | 0.000 | 0.000 |

3 A3. Forehead

| Parameter | *p*-value | |
| --- | --- | --- |
|  | 2 weeks | 4 weeks |
| 1) Average depth of wrinkles | 0.004 | 0.000 |
| 2) Mean depth biggest wrinkle | 0.001 | 0.000 |
| 3) Max depth biggest wrinkle | 0.008 | 0.000 |
| 4) Total wrinkle count | 0.002 | 0.000 |
| 5) Total wrinkle volume | 0.008 | 0.000 |
| 6) Total wrinkle area | 0.001 | 0.000 |
| 7) Total length of wrinkles | 0.002 | 0.003 |
| 8) Ra | 0.003 | 0.000 |
| 9) Rz | 0.002 | 0.000 |

3C. Skin Sagging

| *p*-value | |
| --- | --- |
| 2 weeks | 4 weeks |
| 0.000 | 0.000 |

3D. Skin Pigmentation

| *p*-value | |
| --- | --- |
| 2 weeks | 4 weeks |
| 0.000 | 0.000 |

3E. Dermal density

| *p*-value |
| --- |
| 4 weeks |
| 0.000 |

3F. Mechanical imprint(pressure) relief

| *p*-value | | |
| --- | --- | --- |
| 2 weeks | 4 weeks | 2 weeks vs 4 weeks |
| 0.000 | 0.000 | 0.000 |

**Figure 4. Analysis of parameters after 1 week of discontinuation of the product**

4A. Crow’s feet

| Parameter | *p*-value | | |
| --- | --- | --- | --- |
|  | 4 weeks | After 1 week of discontinuation of the product | 4 week vs. After 1 week of discontinuation of the product |
| 1) Ra | 0.048 | 0.045 | 0.620 |
| 2) Rmax | 0.003 | 0.001 | 0.087 |
| 3) Rz | 0.001 | 0.002 | 0.409 |
| 4) Rp | 0.000 | 0.002 | 0.296 |
| 5) Rv | 0.048 | 0.014 | 0.151 |

4C. Skin Pigmentation

| *p*-value | | |
| --- | --- | --- |
| 4 weeks | After 1 week of discontinuation of the product | 4 week vs. After 1 week of discontinuation of the product |
| 0.000 | 0.000 | 0.197 |

**Figure 5. Analysis of parameters after application of the product once**

5A. Fine lines of the eye area

| Parameter | *p*-value |
| --- | --- |
|  | Immediately after application |
| 1) Ra | 0.000 |
| 2) Rmax | 0.000 |
| 3) Rz | 0.000 |
| 4) Rp | 0.000 |
| 5) Rv | 0.000 |

5C. Skin Sagging

| *p*-value |
| --- |
| Immediately after application |
| 0.000 |

5D. Skin Gloss

| *p*-value |
| --- |
| Immediately after application |
| 0.000 |

5E. Skin Hydration

| *p*-value |
| --- |
| Immediately after application |
| 0.000 |
