# Supplementary material for: The antiaging effects of a product containing collagen and ascorbic acid: In vitro, ex vivo, and pre-post intervention clinical trial
Source: PLoS One. 2022 Dec 12;17(12):e0277188. doi: 10.1371/journal.pone.0277188 (PMC9744321; doi:10.1371/journal.pone.0277188)
Supplement: S2 Table — (DOCX) [file pone.0277188.s003.docx]

**S2 Table.** **Formula for Safety Evaluation**

| Score | Skin Irritation | | | | | | | |
| --- | --- | --- | --- | --- | --- | --- | --- | --- |
| 0 | No sign of inflammation; normal skin | | | | | | | |
| 0.5 | Glazed appearance of the sites, or barely perceptible erythema | | | | | | | |
| 1 | Slight erythema | | | | | | | |
| 2 | Moderate erythema, possibly with barely perceptible edema at the margin, papules may be present | | | | | | | |
| 3 | Moderate erythema, with generalized edema | | | | | | | |
| 4 | Severe erythema with severe edema, with or without vesicles | | | | | | | |
| 5 | Severe reaction spread beyond the area of the patch | | | | | | | |
| Irritation Score  (IS) | | | = | | ∑ (Score × No. of Responders) | x 100 | |  |
|  |  |  |  |  | 4 (Maximum Score) x N (Total Subjects) |  |  |  |
| Mean of Irritation Score  (MIS) | | = | | (Irritation Score after 30 minutes Patch Removal  + Irritation Score after 24 hours Patch Removal) | | |  |  |
|  |  |  |  | 2 (Time Point) | | |  |  |
