# Supplementary material for: The antiaging effects of a product containing collagen and ascorbic acid: In vitro, ex vivo, and pre-post intervention clinical trial
Source: PLoS One. 2022 Dec 12;17(12):e0277188. doi: 10.1371/journal.pone.0277188 (PMC9744321; doi:10.1371/journal.pone.0277188)
Supplement: S1 Text — (DOCX) [file pone.0277188.s004.docx]

**S1 Text. Skin Primary Irritation procedure**

**STUDY PROCEDURE:**

1. The test region (back skin) was washed with 70% ethanol and dried.

2. “Olivapep-Wr” (20 µL) was distributed in IQ Ultra^TM^ Patch Test Units (Chemotechnique Diagnostics AB, Sweden).

3. The IQ chamber was attached to the test site for 24 hours.

4. After removing the patch and marking the test region with a skin marker (skin marker pen, DeRoyeal, USA), each test region was assessed under a magnifying lens (BD -FS3, ARO, Korea) at 30 min and 24 h after the removal of the patch.

Skin irritation potential was evaluated according to the PCPC guidelines (Table 1).

| Irritation Score  (IS) | = | ∑ (Score × No. of responders) | × 100 | |
| --- | --- | --- | --- | --- |
|  |  | 4 (Maximum score) × N (Total No. of subjects) |  |  |
| Mean of Irritation Score  (MIS) | = | (Irritation score after 30 minutes of patch removal  + Irritation score after 24 hours of patch removal) | |  |
|  |  | 2 (Time point) | |  |

The irritation score was derived using the formula given above, and the result was inferred based on the criteria listed in Table 2.

Table 1. Skin Irritation Scoring System

| Score | Skin Irritation |
| --- | --- |
| 0 | No sign of inflammation; normal skin |
| 0.5 | Glazed appearance of the sites, or barely perceptible erythema |
| 1 | Slight erythema |
| 2 | Moderate erythema, possibly with barely perceptible edema at the margin, papules may be present |
| 3 | Moderate erythema, with generalized edema |
| 4 | Severe erythema with severe edema, with or without vesicles |
| 5 | Severe reaction spread beyond the area of the patch |

Table 2. Determination Criteria for Skin Primary Irritation Potential

| Range of Irritation | Determination |
| --- | --- |
| 0.00 ≤ MIS < 0.87 | None to slight |
| 0.87 ≤ MIS < 2.42 | Mild |
| 2.42 ≤ MIS < 3.44 | Moderate |
| 3.44 ≤ MIS | Severe |

*Ref.: International Journal of Cosmetic Science 2014, 36(1), 62-67 ^8)^*
