# Supplementary material for: The antiaging effects of a product containing collagen and ascorbic acid: In vitro, ex vivo, and pre-post intervention clinical trial
Source: PLoS One. 2022 Dec 12;17(12):e0277188. doi: 10.1371/journal.pone.0277188 (PMC9744321; doi:10.1371/journal.pone.0277188)
Supplement: S1 File — (DOCX) [file pone.0277188.s005.docx]

**STANDARD PROTOCOL**

| **A CLINICAL STUDY TO GENERAL EVALUATION**  **PRO-GE-022** |
| --- |

**
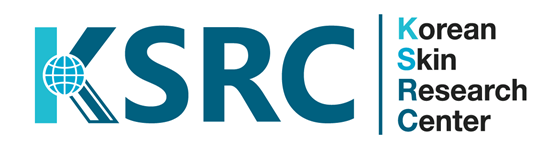
**

**TABLE OF CONTENTS**

1. **STUDY PURPOSE --------------------------------------------------------------------------------3**
2. **RESEARCH INSTITUTION --------------------------------------------------------------------3**
3. **PRINCIPAL INVESTIGATOR AND INVESTIGATOR ----------------------------------3**
4. **CLINICAL STUDY GUIDELINES-------------------------------------------------------------3**
5. **STUDY SUBJECTS ------------------------------------------------------------------------------5**
6. **STUDY PROCEDURE ---------------------------------------------------------------------------7**
7. **ADVERSE EVENT HANDLING GUIDELINES -----------------------------------------10**
8. **STATISTICAL ANALYSIS --------------------------------------------------------------------10**
9. **REFERENCES -----------------------------------------------------------------------------------12**

**Ⅰ. STUDY PURPOSE**

This study was conducted to evaluate the skin folds (Crow’s feet, forehead, and nasolabial folds), skin pigmentation, sagging, dermal density, gloss, mechanical imprint(pressure) relief, skin surface to a depth of 2.5mm hydration and skin safety of the test product for the human skin.

**Ⅱ. RESEARCH INSTITUTION**

1. Research Institution: KSRC Korean Skin Research Center
2. Tel : 031-712-8520
3. Email: ksrc@koreansrc.com
4. Address: 24F, 8, Seongnam-daero 331beon-gil, Bundang-gu, Seongnam-si, Gyeonggi-do, Korea

**Ⅲ. PRINCIPAL INVESTIGATOR AND INVESTIGATOR**

1. Principal Investigator: So Min Kang, Ph.D.
2. Investigator: Taekyeong Ryu, Hanna Lee, Dayeong Nam, Sooyun Lee, Byung Ho Shin, Gowoon Choi, Dasom Jeon, Bobae Oh, Jihyun Kim, Young Yoon, Hyunjeong Kim

**Ⅳ. CLINICAL STUDY GUIDELINES**

This study was conducted according to GCP (Good Clinical Practice), MFDS (Ministry of Food and Drug Safety) and SOP (Standard Operating Procedures) of KSRC CO., LTD.

**Ⅴ. STUDY SUBJECTS**

In this study, the purpose and method, expected efficacy and side effects of this study are explained by selecting those who meet the selection criteria and not the exclusion criteria. Research subjects who show their intention to participate should fill out a consent form for research participation and participate in this study.

**Ⅴ-I. Inclusion Criteria**

1. Korean female volunteers aged 40-59 years with skin folds (eye corners, forehead, nasolabial folds) and pigmentation on the face
2. Healthy subjects free from acute and chronic diseases including skin conditions
3. Subjects who had voluntarily signed the informed consent form after understanding the complete explanation of the purpose and protocol of this study
4. Subjects who are available for follow up during the study period
5. Subjects who are not hypersensitive to vitamin C

**Ⅴ-II. Exclusion Criteria**

1. Women who is pregnant, lactating or planning to become pregnant within 6 months
2. Subjects who have skin diseases, including active atopic dermatitis, psoriasis, eczema and active seasonal allergies on the test region
3. Subjects who have used antibacterial agents, immunosuppressants, external skin prepara-tions containing steroids and treatments for chronic skin conditions for more than 1 month to treat skin conditions on the test region
4. Subjects who have not passed 1 month since participating in the same study
5. Subjects who used the same or similar efficacy cosmetics and medicines on the test site within 3 months prior to the start of the study
6. Subjects with chronic diseases (asthma, diabetes, hypertension, etc.)
7. Subjects on contraceptives, antihistamines and anti-inflammatory drugs
8. Subjects who are employees in this clinical research institute
9. Subjects who were considered as inappropriate according to the judgment of the investigator

**V-IV.** **Number of Subjects**

This study recruited over 20 subjects according to the recommendations of MFDS Guideline.

**Ⅵ. STUDY PROCEDURE**

In this study, all evaluations were conducted via the same site measurement. The study subjects participated after washing the test site during the visit and stabilizing for 30 min in a constant temperature and humidity room (22 ± 2°C, 50 ± 5%).

**Ⅵ-I. Photography**

In this study, the facial image was taken in optical and polarized mode using VISIA^®^ CR (Canfield, USA) at baseline, and immediately after test product application (Figure 1).


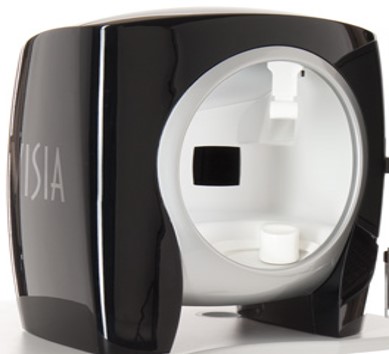

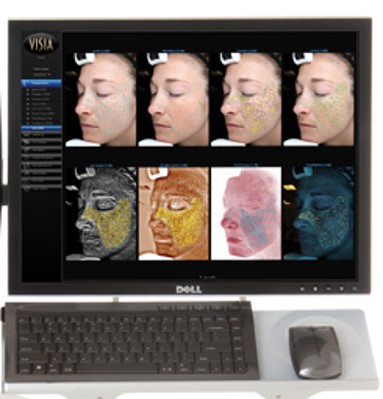


Figure 1. VISIA^®^ CR 2.3

**Ⅵ-Ⅱ. Measurement of wrinkle**

The Primos-CR (Canfield Scientific, Parsippany-Troy Hills, New Jersey) was used to evaluate the three-dimensional surface of the skin. Data files captured by the Primos-CR were analyzed using the Primos software (Figure 2).


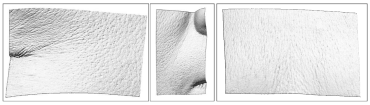


Figure 2. PRIMOS^CR^ SF and 3D image of the Crow’s feet, Nasolabial fold and forehead area

Table 1. Skin wrinkle parameters

| **Parameter** | |
| --- | --- |
| (1) Average depth of wrinkles (㎛) | |
| (2) Mean depth biggest wrinkle (㎛) | |
| (3) Max. depth biggest wrinkle (㎛) | |
| (4) Total wrinkle count (Number) | |
| (5) Total wrinkle volume (mm^3^) | |
| (6) Total wrinkle area (mm^3^) | |
| (7) Total length of wrinkles (mm^3^) | |
| (8) Ra (Arithmetic average) (㎛) | 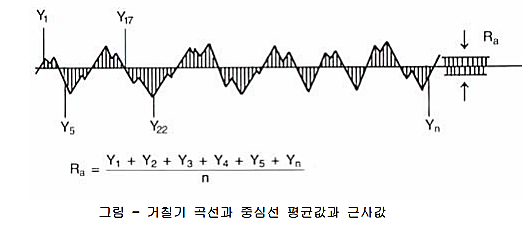 |
| (9) Rz (Average maximum height of the profile) (㎛) | 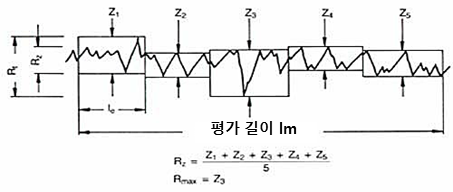 |

**Ⅵ-Ⅲ. Measurement of Skin Pigmentation**

The skin color of the cheek of volunteers was measured using a CM-26dG spectrophotometer (Konica Minolta, INC., Osaka, Japan). The L* value express the relative brightness follow CIE (Commission Internationale de l’Eclairage) L*a*b* system (Figure 3).


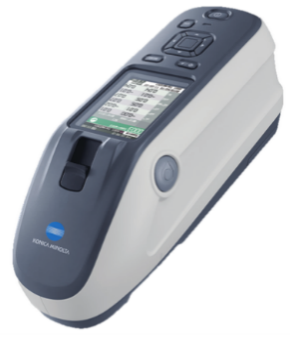

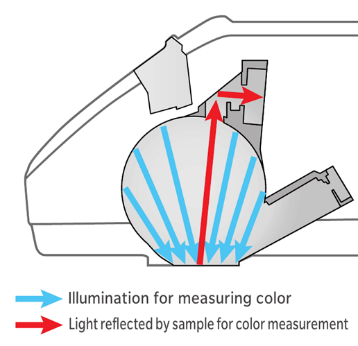

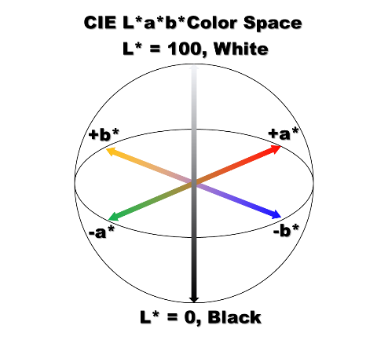


Figure 3. Image and measurement principle of Spectrophotometer^®^ CM26dG)

**Ⅵ-Ⅳ. Measurement of Skin sagging**

F-RAY (BEYOUNG, Korea) images of facial contour curves of the selected cheek area were taken before and immediately after product use. The angle of the contour line was analyzed for the captured contour image using Image Pro^®^ 10 (Media Cybernetics, USA), an analysis program (Figure 4).


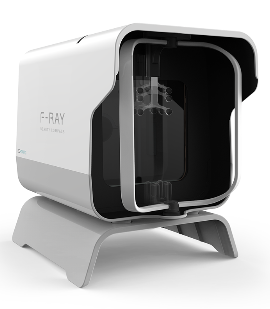

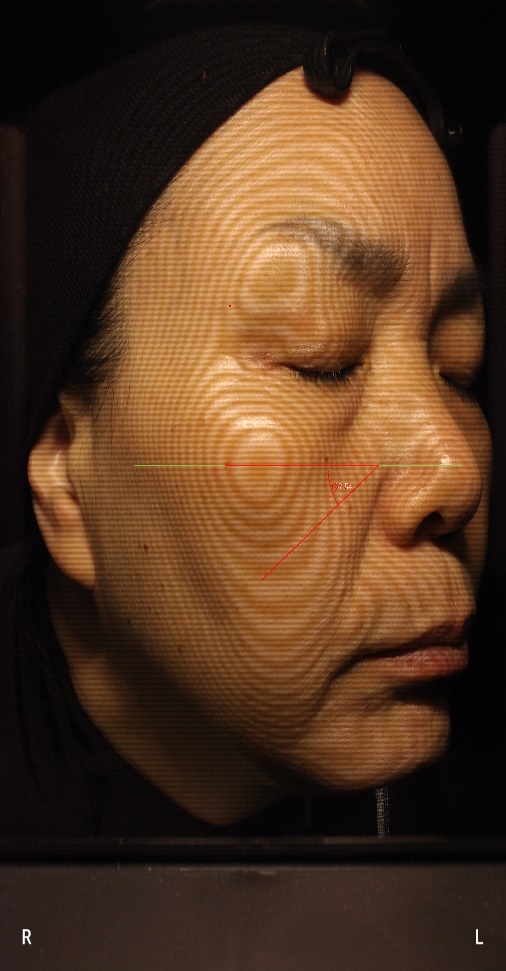


Figure 4. F-RAY and Analysis example image

**Ⅵ-Ⅴ. Measurement of dermal density**

The skin dermal density of the cheek of volunteers was measured using the Ultrasound Probe of DermaLab® Series SkinLab Combo (Cortex Technology, Denmark). The low density was displayed as a dark color and high density is displayed as a bright color through the signal strength (Figure 5).


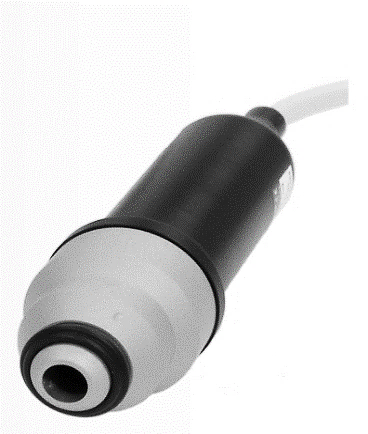

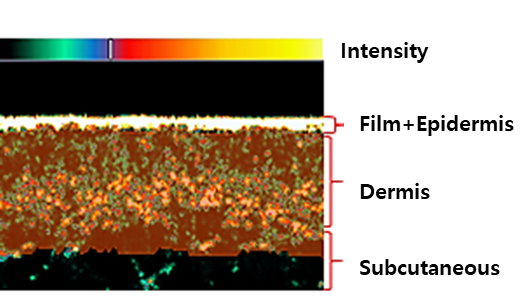


Figure 5. Ultrasound probe and analysis example image

**Ⅵ-Ⅳ. Measurement of mechanical imprint (pressure) relief**

The skin pressure impression of the cheek of volunteers made by applying physical force to uneven cotton (8room, Korea). The Primos-CR was used to evaluate the surface of the skin. The image captured by the Primos-CR were analyzed using the Primos software (Figure 6).


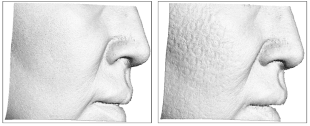


Figure 6. Analysis of mechanical imprint (pressure) relief 3D image

Table 4. Skin texture (roughness) parameters

| **Parameter** | **Definition** |
| --- | --- |
| Rmax | Maximum peak to valley roughness height (㎛) |

**Ⅵ-Ⅶ. Measurement of skin gloss (radiance)**

Mark-Vu (Mark-Vu^®^; PSI PLUS Co., Ltd., Suwon, Korea), a skin diagnostic imaging system, was used to analyze the skin gloss. The analysis was performed in the designated area on the polarized image using the Image-Pro^®^ 10 (Media Cybernetics, USA) program (Figure 7).


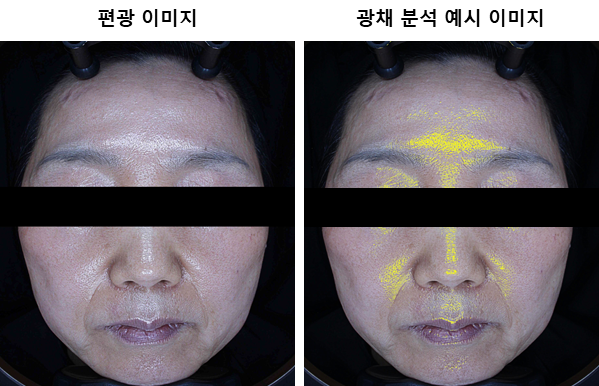


Figure 7. Anaylsis of skin gloss (radiance) example image

**Ⅳ-Ⅷ. Measurement of hydration in the stratum corneum**

Dermal hydration was measured dielectrically with an open-ended coaxial probe that was layered in structure (Moisture Meter-D; Delphin Technologies Ltd.) (Figure 8).


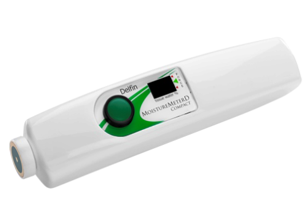

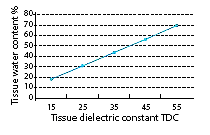


FIgure 8. MoistureMeter D Compact and analysis example image

**Ⅵ-Ⅸ. Safety Evaluation**

As concerns the safety assessment, the researchers evaluated subjective and objective signs through clinical observations. In the event of an adverse reaction, an adverse reaction report was prepared and the principal investigator determined the relationship with the test product.

**Ⅶ. ADVERSE EVENT HANDLING GUIDELINES**

During the study period, the researcher observes the skin condition of the research subjects and, if serious adverse reactions occur other than predictable adverse reactions, prompt and appropriate measures are taken to minimize possible adverse reactions.

In the event of an adverse reaction, the research director decides on the relevance to the test product, and if a problem occurs due to the test product, the sponsoring institution takes full responsibility, and ensures the safety of the research subject by making appropriate compensation.

**Ⅷ. STATISTICAL ANALYSIS**

1. Statistical analysis was done using the SPSS® software program (IBM, USA).
2. The normality test was verified through the Shapiro–Wilk test and kurtosis & skewness.
3. Statistical analysis of variables for parametric values was performed using the paired t-test and RM-ANOVA. *p* values <0.05 were considered significant and indicated by * and *p* values <0.001 were considered significant and indicated by ***.

**Ⅸ. REFERENCE**

1. MFDS. Partial Revision Notice of Regulations on the Examination of Functional Cosmetics. **2019-47**.
2. MFDS. Regulations on the Demonstration of Labeling and Advertisement for Cosmetic Products. **2018.03**.
3. MFDS. Guidelines for Human Application Test and Efficacy Test for Cosmetics. **2015.**
4. Peperkamp, K.; Verhulst, A. C.; Tielemans, H. J.; Winters, H.; van Dalen, D.; Ulrich, D. J., inter‐rater and test‐retest reliability of skin thickness and skin elasticity measurements by the DermaLab Combo in healthy participants. *Skin Research and Technology* **2019,** *25*(6), 787-792.
5. Hadi, H.; Awadh, A. I.; Hanif, N. M.; Md Sidik, N. F. A.; Mohd Rani, M. R. N.; Suhaimi, M. S. M., The investigation of the skin biophysical measurements focusing on daily activities, skin care habits, and gender differences. *Skin Research and Technology* **2016,** *22*(2), 247-254.
6. Frosch, P. J.; Kligman, A. M., Noninvasive methods for the quantification of skin functions: An update on methodology and clinical applications. *Springer Science & Business Media*: **2012**.
7. Tsukahara, K.; Sugata, K.; Osanai, O.; Ohuchi, A.; Miyauchi, Y.; Takizawa, M.; Hotta, M.; Kitahara, T., Comparison of age-related changes in facial wrinkles and sagging in the skin of Japanese, Chinese and Thai women. *Journal of dermatological science* **2007,** *47* (1), 19-28.
8. Callaghan, T.; Wilhelm, K. P., A review of ageing and an examination of clinical methods in the assessment of ageing skin. Part 2: Clinical perspectives and clinical methods in the evaluation of ageing skin. *International journal of cosmetic science* **2008,** *30* (5), 323-332.
9. Alaluf, S.; Atkins, D.; Barrett, K.; Blount, M.; Carter, N.; Heath, A., The impact of epidermal melanin on objective measurements of human skin colour. *Pigment cell research* **2002.**
10. Harding, C.; Watkinson, A.; Rawlings, A.; Scott, I. J. I. j. o. c. s., Dry skin, moisturization and corneodesmolysis. **2000,** 22 (1), 21-52.
11. Fischer, T. W.; Wigger-Alberti, W.; Elsner, P., Direct and non-direct measurement techniques for analysis of skin surface topography. *Skin Pharmacology and Physiology* **1999,** *12* (1-2), 1-11.
12. Piérard, G., EEMCO guidance for the assessment of skin colour. *Journal of the European Academy of Dermatology and Venereology* **1998,** *10* (1), 1-11.
13. Berardesca, E.; Cosmetics, E. G. f. E. M. o.; Products, O. T., EEMCO guidance for the assessment of stratum corneum hydration: electrical methods. *Skin Research and Technology* **1997,** *3* (2), 126-132.
14. Grove, G. J. C. t. r., The effect of moisturizers on skin surface hydration as measured in vivo by electrical conductivity. **1991,** 50 (5), 712-719.
15. DANIELL, H. W., Smoker's wrinkles: a study in the epidemiology of "crow's feet". *Annals of internal medicine* **1971,** *75* (6), 873-880.
