# Supplementary material for: The antiaging effects of a product containing collagen and ascorbic acid: In vitro, ex vivo, and pre-post intervention clinical trial
Source: PLoS One. 2022 Dec 12;17(12):e0277188. doi: 10.1371/journal.pone.0277188 (PMC9744321; doi:10.1371/journal.pone.0277188)
Supplement: S2 File — (DOCX) [file pone.0277188.s006.docx]

**STANDARD PROTOCOL**

| **인체피부 일반 생리 평가**  **PRO-GE-022** |
| --- |

**한국피부임상연구센터(KSRC)**

**목 차**

1. 연구 목적 -------------------------------------------------------------------------------------------3
2. 연구 기관 -------------------------------------------------------------------------------------------3
3. 연구 책임자 및 담당 연구자 ----------------------------------------------------------------------3
4. 인체적용시험 지침 ---------------------------------------------------------------------------------3
5. 연구대상자 ------------------------------------------------------------------------------------------5
6. 연구방법 --------------------------------------------------------------------------------------------7
7. 이상반응 처리 지침 -------------------------------------------------------------------------------10
8. 통계분석 -------------------------------------------------------------------------------------------10
9. 참고문헌 ------------------------------------------------------------------------------------------12

**1. 연구 목적**

본 연구는 인체피부에서 시험제품에 대한 3대 주름(눈꼬리, 팔자, 이마) 및 미세주름, 피부 색, 리프팅, 치밀도, 모공, 광채, 안면 베개 자국 완화 및 피부 속(2.5 mm) 수분량 개선효과 및 피부 안전성을 평가하기 위함이다.

**2. 연구 기관**

1. 기관명: KSRC 한국피부임상연구센터
2. 연락처: 031-712-8520
3. 이메일: ksrc@koreansrc.com
4. 주소: 경기도 성남시 분당구 성남대로 331번길 8

**3. 연구 책임자 및 담당 연구자**

1. 연구책임자: 허찬영 성형외과 전문의
2. 공동연구자: 류태경, 이한나, 남다영, 이수연, 신병호, 최고운, 전다솜, 오보배, 김지현, 윤영,

김현정 연구원

**4. 인체적용시험 지침**

본 연구는 GCP (Good Clinical Practice), MFDS (Ministry of Food and Drug Safety) 관련규정 및 한국피부임상연구센터(KSRC)의 표준작업지침서(SOP)에 규정 및 조항을 준수하도록 한다.

**5. 연구 대상자**

본 연구에서는 연구대상자의 선정기준에 부합하고 제외기준에 부합하지 않는 자를 선정하여 본 연구의 목적과 방법, 기대효능과 부작용을 설명한다. 참여 의사를 보이는 연구대상자는 연구 참가 동의서를 작성하고 본 연구에 참여하도록 한다.

5-1. 대상자 선정기준

1. 눈가 주름이 있는 40~59세의 여성 지원자
2. 피부 질환을 포함하는 급, 만성 신체 질환이 없는 건강한 지원자
3. 연구 참여에 대하여 충분히 설명을 듣고 자발적으로 연구 참여 동의서를 작성하고 서명한 지원자
4. 연구기간 동안 추적 관찰이 가능한 지원자
5. 비타민 C에 과민 반응이 없는 지원자

5-2. 대상자 제외 기준

1. 임신, 수유 중 또는 6개월 이내에 임신을 계획하고 있는 지원자
2. 연구부위에 피부질환(심한 염증, 습진, 건선, 피부암 등), 피부알러지, 민감성, 과민성 피부를 가지고 있는 지원자
3. 연구부위에 피부질환의 치료를 위해 항균제, 면역억제제, 스테로이드가 함유된 피부 외용제 및 만성피부질환 치료제를 1개월 이상 사용하고 있는 지원자
4. 동일한 연구에 참가한 뒤 3개월이 경과되지 않은 지원자
5. 연구 시작 전 3개월 내 연구부위에 동일 또는 유사한 효능의 화장품, 의약품 등을 사용한 지 원자
6. 만성 소모성 질환이 있는 지원자(천식, 당뇨, 고혈압 등)
7. 피임제, 항히스타민제, 소염제를 복용하고 있는 지원자
8. 본 임상연구소의 임직원인 지원자
9. 그 외 연구담당자의 판단으로 연구가 부적합하다고 판단되는 지원자

5-3. 연구대상자 수 산출기준

본 연구는 결과 데이터의 통계적 유효성 비교분석을 위한 최소 연구대상자 수인 20명 이상의 지 원자를 모집하여 연구를 실시하였다(기능성화장품의 유효성평가를 위한 가이드라인, 화장품 표시, 광고 실증을 위한 시험방법 가이드라인).

**7. 연구 방법**

본 연구에서는 각 평가시점에서 연구대상자의 측정부위를 선정하고, 모든 평가는 동일 부위 측정 을 통해 진행한다.

6-1. 사진촬영

본 연구에서는 각 평가시점에서 연구대상자의 자세, 거리, 조도 등이 동일한 조건이 되도록 고정한 다음 안면촬영장치인 VISIA^®^ CR 2.3(Canfield, USA)를 이용하여 안면 좌, 우 측면을 광학 및 편광 모드로 촬영한다(그림 1).


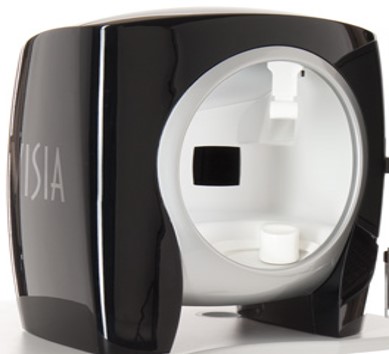

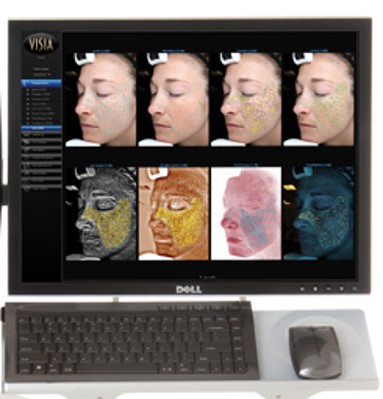


그림 1. VISIA^®^ CR 2.3 기기 이미지

6-2. 피부 3대 주름 및 미세주름(눈꼬리) 측정

본 연구에서는 각 평가시점에서 선정된 3대 주름(눈꼬리, 팔자, 이마) 및 미세주름(눈꼬리) 부위의 3D 이미지를 촬영하고 분석 프로그램을 이용하여 피부 주름 및 거칠기(표 1, 2) 파라미터 값을 분 석하였다.

피부 주름 측정은 고해상도 센서를 이용한 3차원 이미징 시스템 PRIMOS CR SF (Canfield, USA)을 이용하였다(그림 2).


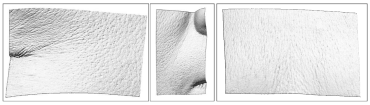


그림 2. 눈꼬리, 팔자 및 이마 주름 3D 예시 이미지

표 1. 피부 주름 분석 파라미터

| **파라미터** | **정의** | |
| --- | --- | --- |
| (1) Average depth of wrinkles | | 평균 주름 깊이(㎛) |
| (2) Mean depth biggest wrinkle | | 가장 큰 주름의 평균적인 깊이(㎛) |
| (3) Max. depth biggest wrinkle | | 가장 큰 주름의 최대 깊이(㎛) |
| (4) Total wrinkle count | | 주름 수(Number) |
| (5) Total wrinkle volume | | 주름 부피의 합계(mm^3^) |
| (6) Total wrinkle area | | 주름 면적의 합계(mm^2^) |
| (7) Total length of wrinkles | | 전체 주름의 길이(mm) |
| (8) Ra | | 산술 평균 거칠기(㎛)  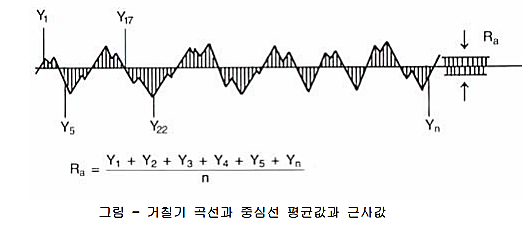 |
| (9) Rz | | 10점 평균 거칠기(㎛)  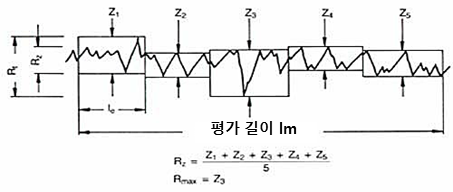 |

표 2. 피부 거칠기 분석 파라미터

| **파라미터** | **정의** |
| --- | --- |
| (1) Ra (Arithmetic average) | 산술 평균치(㎛) |
| (2) Rmax (Maximum peak to vally roughness height) | 최대 거칠기(㎛) |
| (3) Rz (Average maximum height of the profile) | 10점 평균 거칠기(㎛) |
| (4) Rp (Largest positive deviation) | 최대 단면 산 높이(㎛) |
| (5) Rv (Largest negative deviation) | 최대 단면 골 높이(㎛) |

6-3. 피부 색 측정

본 연구에서는 각 평가시점에서 선정된 과색소침착 부위의 피부 색(L* value)을 3회 측정하여 평균 값을 분석한다.

피부 색을 측정할 수 있는 Spectrophotometer^®^ CM26dG (Minolta, Japan)의 광학계는 적분구를 탑재한 확산 조명 8° 수광 방식으로 시료의 분광반사율 및 Tristimulus values를 측정하여 CIE L*a*b*의 표색계를 따른다(그림 3). L*a*b* 표색계에서 명도는 L*(밝기)로 표시하고, a*, b*는 색상과 채도를 색의 방향을 표시하고 있다(a*: 적색, –a*: 녹색, b*: 황색, -b*: 청색 방향).


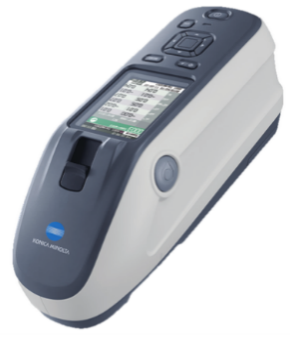

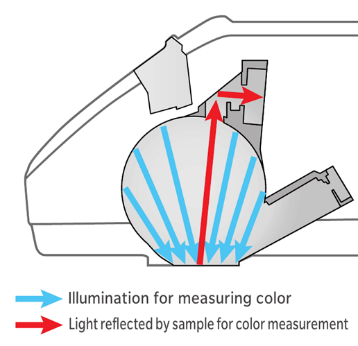

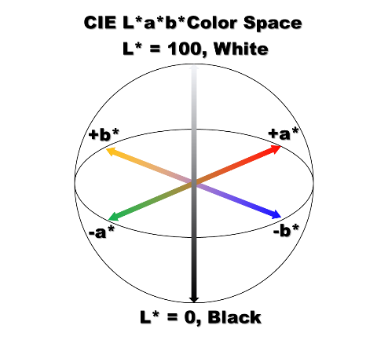


그림 3. Spectrophotometer^®^ CM26dG 기기 및 측정 원리

(왼쪽부터 Spectrophotometer^®^ CM26dG, 적분구 수광 방식, CIE L*a*b* 표색계)

6-4. 피부 리프팅 측정

본 연구에서는 각 평가시점에서 선정된 뺨 부위의 피부 등고선(굴곡) 이미지를 F-RAY (BEYOUNG, Korea)로 촬영하였다.

본 측정기기는 일정한 간격을 갖는 무늬가 반복되어 겹쳐져 나타나는 모아레(Moire) 현상을 통 해 얼굴 표면의 고저, 윤곽, 주름과 꺼짐 등을 등고선 이미지로 나타낸다. 촬영된 이미지는 분석 프로그램인 Image-Pro^®^ 10 (Media Cybernetics, USA)을 이용하여 등고선의 각도를 측정함으로써 분석되었다(그림 4).


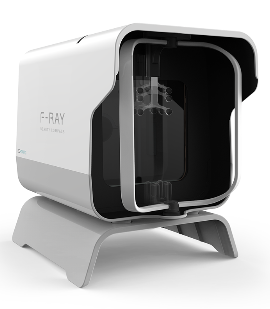

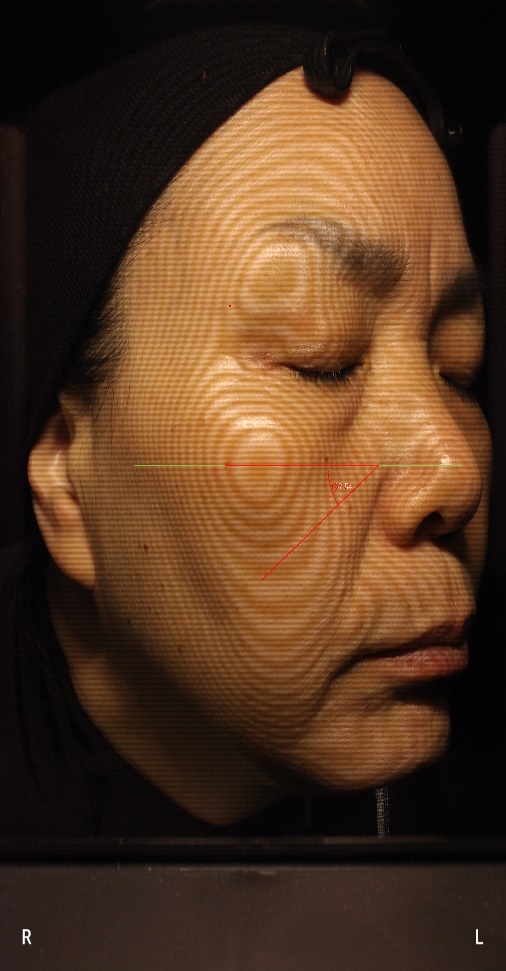


그림 4. F-RAY 기기 및 분석 예시 이미지

6-5. 피부 치밀도 측정

본 연구에서는 각 평가시점에서 선정된 뺨 부위의 피부 치밀도를 DermaLab^®^ Series SkinLab Combo (Cortex Technology, Denmark)의 Ultrasound probe를 이용하여 측정한다.

본 측정기기는 초음파 이미징 기기로 피부에 음향 펄스를 투사시킴으로 그 반응도를 측정하여 신호의 강도를 통해 낮은 치밀도는 어두운 색상으로서 높은 치밀도는 밝은 색상으로서 출력하여 나타낸다(그림 5).


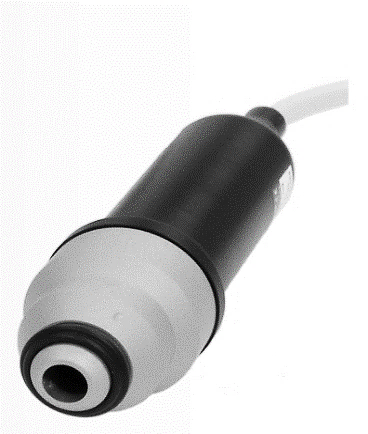

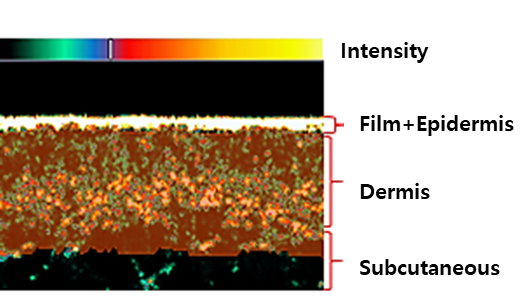


그림 5. Ultrasound probe 및 분석 이미지

6-6. 안면 베개 자국 측정

본 연구에서는 제품 사용에 의한 안면 베개 자국 완화 효과를 평가하기 위하여 제품 사용 전 및 제품 사용 4주 후 시점에서 선정된 뺨 부위에 10분 동안 인위적으로 베개 자국을 유도하였다.

베개 자국 유도 전, 제품 사용 전 및 제품 사용 4주 후 시점에서 베개 자국이 유도된 뺨 부위의 3D 이미지 를 촬영하고 분석 프로그램을 이용하여 거칠기(표 4) 파라미터 값을 분석하였다.

안면 베개 자국 측정은 고해상도 센서를 이용한 3차원 이미징 시스템 PRIMOS CR LF (Canfield, USA)을 이용하였다(그림 6).


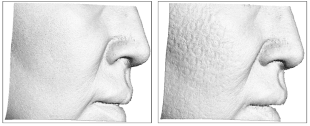


그림 6. 안면 베개 자국 유도 전 및 후 3D 예시 이미지

표 4. 피부 거칠기 분석 파라미터

| **파라미터** | **정의** |
| --- | --- |
| Rmax (Maximum peak to vally roughness height) | 최대 거칠기(㎛) |

6-7. 피부 광채 측정

본 연구에서는 제품 사용 전 및 제품 사용 직후 시점에서 안면 측정 기기인 MARK.Vu (PSIPlus, Korea)를 이용하여 연구대상자의 안면을 촬영하였고, Image-Pro^®^ 10 (Media Cybernetics, USA) 프로 그램을 이용하여 분석을 진행하였다.

피부 광채는 편광 이미지 상의 지정한 분석 영역에서 명도 값(Intensity value)의 평균값을 분석하 였다(그림 7).


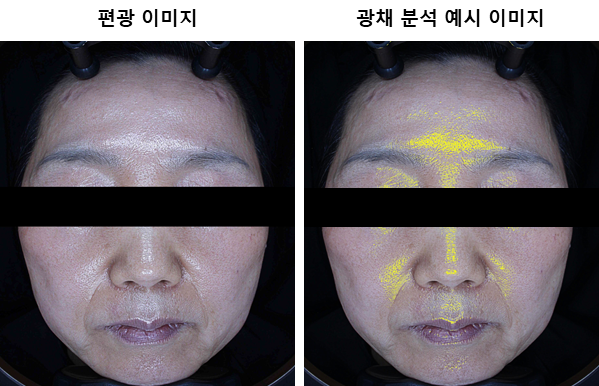


그림 7. 피부 광채 분석 예시 이미지

6-8. 피부 속 수분량 측정

본 연구에서는 제품 사용 전 및 제품 사용 직후 시점에서 선정된 뺨 부위의 피부 속(2.5 mm) 수분량을 MoistureMeter D Compact (Delfin, Finland)를 이용하여 3회씩 측정한 후 평균값을 분석하 였다.

본 측정기기는 비침습적 방법으로 표피와 진피 사이의 조직 유전 상수(tissue dielectric constant, TDC)를 이용하여 피부 조직내 2.5 mm 깊이의 수분량 변화를 0~100% 단위로 변환하여 나타낸다. 또한, 접촉형 압력센서가 내장되어 있어 매 측정 시 동일한 조건에서의 측정이 가능하며, 측정되는 피부 깊이는 probe 직경에 따라 달라지고 측정값은 수분량에 비례한다(그림 8).


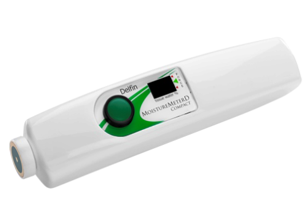

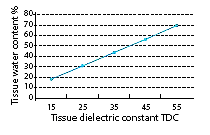


그림 8. MoistureMeter D Compact 기기 및 측정 원리 이미지

6-10. 피부 이상반응 평가

안전성 평가를 위해 연구자는 각 평가시점에서 연구대상자의 시험 부위를 관찰한 후 연구대상자와 의 질의 응답을 통해 시험 부위 상태를 확인하여 기록, 평가하였다. 제품으로 인한 이상반응 발생 시 이상반응 보고서를 작성하도록 하였으며, 이에 대한 시험제품과의 관련성은 연구책임자가 판단 한다.

**7. 이상반응 처리 지침**

연구기간 동안 연구자가 연구대상자의 피부 상태를 관찰하고 예측 가능한 이상반응 이외에 심각한 이상반응 발생 시 신속하고 적절한 조치를 취하여 가능한 그 이상반응을 최소화한다.

이상반응 발생 시 이에 대한 시험제품과의 관련성에 관해서는 연구책임자가 판단하며, 시험 제품으로 인하여 문제가 발생되는 경우 의뢰기관에서 전적으로 책임지며, 그에 대한 적절한 보상을 하도록 하여 연구대상자의 안전에 만전을 기한다.

**8. 통계 분석**

1. 산출된 모든 데이터는 SPSS Package Program ver. 20 (IBM, USA)을 이용하여 통계적 유의성을 검증한다.
2. 데이터의 정규성은 Shapiro-Wilk test 와 첨도/왜도(Kurtosis&skewness)를 통해 검증한다.
3. 모든 평가결과에 대한 전후 비교는 Paired *t*-test 및 RM-ANOVA를 이용한다(*p*<0.05).
4. 전후 변화에 따른 변화율은 다음과 같이 산출한다.

| 변화율(%) = | │제품 사용 전 – 제품 사용 후│ | x 100 |
| --- | --- | --- |
|  | 제품 사용 전 |  |

**9. 참고 문헌**

1. MFDS. 기능성 화장품 심사에 관한 규정 일부 개정고시. **제2019-47**.
2. MFDS. 화장품 표시·광고 실증을 위한 시험방법 가이드라인. **2018.03**.
3. MFDS. 화장품 인체적용시험 및 효력시험 가이드라인. **2015.**
4. Peperkamp, K.; Verhulst, A. C.; Tielemans, H. J.; Winters, H.; van Dalen, D.; Ulrich, D. J., inter‐rater and test‐retest reliability of skin thickness and skin elasticity measurements by the DermaLab Combo in healthy participants. *Skin Research and Technology* **2019,** *25*(6), 787-792.
5. Hadi, H.; Awadh, A. I.; Hanif, N. M.; Md Sidik, N. F. A.; Mohd Rani, M. R. N.; Suhaimi, M. S. M., The investigation of the skin biophysical measurements focusing on daily activities, skin care habits, and gender differences. *Skin Research and Technology* **2016,** *22*(2), 247-254.
6. Frosch, P. J.; Kligman, A. M., Noninvasive methods for the quantification of skin functions: An update on methodology and clinical applications. *Springer Science & Business Media*: **2012**.
7. Tsukahara, K.; Sugata, K.; Osanai, O.; Ohuchi, A.; Miyauchi, Y.; Takizawa, M.; Hotta, M.; Kitahara, T., Comparison of age-related changes in facial wrinkles and sagging in the skin of Japanese, Chinese and Thai women. *Journal of dermatological science* **2007,** *47* (1), 19-28.
8. Callaghan, T.; Wilhelm, K. P., A review of ageing and an examination of clinical methods in the assessment of ageing skin. Part 2: Clinical perspectives and clinical methods in the evaluation of ageing skin. *International journal of cosmetic science* **2008,** *30* (5), 323-332.
9. Alaluf, S.; Atkins, D.; Barrett, K.; Blount, M.; Carter, N.; Heath, A., The impact of epidermal melanin on objective measurements of human skin colour. *Pigment cell research* **2002.**
10. Harding, C.; Watkinson, A.; Rawlings, A.; Scott, I. J. I. j. o. c. s., Dry skin, moisturization and corneodesmolysis. **2000,** 22 (1), 21-52.
11. Fischer, T. W.; Wigger-Alberti, W.; Elsner, P., Direct and non-direct measurement techniques for analysis of skin surface topography. *Skin Pharmacology and Physiology* **1999,** *12* (1-2), 1-11.
12. Piérard, G., EEMCO guidance for the assessment of skin colour. *Journal of the European Academy of Dermatology and Venereology* **1998,** *10* (1), 1-11.
13. Berardesca, E.; Cosmetics, E. G. f. E. M. o.; Products, O. T., EEMCO guidance for the assessment of stratum corneum hydration: electrical methods. *Skin Research and Technology* **1997,** *3* (2), 126-132.
14. Grove, G. J. C. t. r., The effect of moisturizers on skin surface hydration as measured in vivo by electrical conductivity. **1991,** 50 (5), 712-719.
15. DANIELL, H. W., Smoker's wrinkles: a study in the epidemiology of "crow's feet". *Annals of internal medicine* **1971,** *75* (6), 873-880.
