# Supplementary material for: The antiaging effects of a product containing collagen and ascorbic acid: In vitro, ex vivo, and pre-post intervention clinical trial
Source: PLoS One. 2022 Dec 12;17(12):e0277188. doi: 10.1371/journal.pone.0277188 (PMC9744321; doi:10.1371/journal.pone.0277188)
Supplement: Attachment — Submitted filename: PONE-D-22-07546.docx [file pone.0277188.s007.docx]

The manuscript “**The anti-aging effects of the product including both collagen and ascorbic acid: in vitro, ex vivo and pre-post intervention clinical trials**” by Ryu *et.al.* elaborates the use of TEENIALL to enhance the synergetic effect between collagen and ascorbic acid, tested to decrease skin wrinkles and hyperpigmentation in humans within 40 to 59 years. The research had focused on use of TEENIALL in reducing aging effects, with visible results after the first use. These findings should thus motivate further research and would deserve publication. However, I do not think that this paper is publishable in its present form and that at least one round of minor revision is needed.

In the introduction, page 2, the sentence starting with “Ascorbic acid is a representative….” can be given as a separate paragraph.

The products Naticol ® and Quali®-C, are mentioned as “raw materials” in most of the places. Please change that to “products” or any other word of similar meaning.

In the study procedure the sentences” In this study, treatment was applied once, 2 and 4 weeks, the study was conducted as a single test. Over 20 subjects meeting the inclusion and exclusion criteria were enrolled” and “For 4 weeks, the product was applied to subjects on a daily basis around the whole face after washing the face twice a day” are unclear and misleading.

Please correct the grammatical errors in the manuscript. There are several grammatical errors found.

The English language is generally unsatisfactory.

I suggest rewriting the conclusion. The line starting with “In summary….can be corrected”
