# Supplementary material for: The antiaging effects of a product containing collagen and ascorbic acid: In vitro, ex vivo, and pre-post intervention clinical trial
Source: PLoS One. 2022 Dec 12;17(12):e0277188. doi: 10.1371/journal.pone.0277188 (PMC9744321; doi:10.1371/journal.pone.0277188)
Supplement: Attachment — Submitted filename: Response to Reviewers.docx [file pone.0277188.s008.docx]

June 07, 2022

Dr. Parasuraman Padmanabhan

Academic Editor

*PLOS ONE*

Dear Dr. Padmanabhan:

It is with great pleasure that we resubmit the revised version of our manuscript, presently titled “**The antiaging effects of a product containing collagen and ascorbic acid: *In vitro*, *ex vivo*, and pre-post intervention clinical trial,**” by Tae Kyeong Ryu et al. to *PLOS ONE*. The manuscript ID is PONE-D-22-07546R1.

We appreciate the decision of the Editors to reconsider our manuscript and are grateful to the reviewers for their meticulous comments. We have sincerely endeavored to address each comment and have made appropriate modifications to our manuscript. We acknowledge that our manuscript has benefited from the insightful suggestions. We hope that the revised manuscript addresses all the concerns and would be considered suitable for publication in *PLOS ONE*. If you have any further queries, please do not hesitate to contact me.

Thank you for your kind consideration of our manuscript. We look forward to a favorable decision.

Sincerely yours,

So Min Kang, Ph.D.

Korean Skin Research Center (KSRC)

24F, 8, Seongnam-daero 331beon-gil, Bundang-gu, Seongnam-si, Gyeonggi-do, Korea

Fax: +82-70-4467-4499

Email: ksrc@koreansrc.com

**Contents**

**<Reviewer 1>**

**[1] Revision (Major) --------------------------------------------------- 4**

**[2] Revision (Minor) --------------------------------------------------- 8**

**[3] Revision (Minor) ------------------------------------------------- 10**

**[4] Revision (Minor) ------------------------------------------------- 13**

**[5] Revision (Minor) ------------------------------------------------- 16**

**[6] Revision (Minor) ------------------------------------------------- 17**

**<Reviewer 2>**

**[7] Revision (Minor) ------------------------------------------------- 18**

**[8] Revision (Minor) ------------------------------------------------- 19**

**<Reviewer 3>**

**[9] Revision (Minor) ------------------------------------------------- 21**

**[10] Revision (Minor) ------------------------------------------------ 22**

**[11] Revision (Minor) ------------------------------------------------ 23**

**[12] Revision (Minor) ------------------------------------------------ 24**

Major changes and additions in the revised manuscript

We have revised the manuscript and included a number of additional values in supplemental tables.

We marked the edited part of the manuscript with yellow highlight.

**<Reviewer 1>**

**[1] Revision (Major)**
Provide summary tables of results. Include precise *p*-values instead of “*p*<0.05” or “*p*<0.001.”

**Response:** We would like to thank the reviewer for this comment. As suggested, we have provided the exact *p*-values for the data presented in the graphs as supporting information (S1 Table).

**S1 Table.** ***p*-Values for the Graphs Included in the Figures.**

**For data in Figure 2D.** Analysis of collagen synthesis depending on the concentration of the test product.

| Concentrate | 0.01% | 0.02% | 0.05% |
| --- | --- | --- | --- |
| *p*-value | 0.014 | 0.000 | 0.001 |

**For data in Figure 3.** Analysis of parameters after treatment for 4 weeks

3 A1. Crow’s feet

| Parameter | *p*-value | |
| --- | --- | --- |
|  | 2 weeks | 4 weeks |
| 1) Average depth of wrinkles | 0.001 | 0.000 |
| 2) Mean depth biggest wrinkle | 0.000 | 0.000 |
| 3) Max. depth biggest wrinkle | 0.000 | 0.000 |
| 4) Total wrinkle count | 0.029 | 0.022 |
| 5) Total wrinkle volume | 0.000 | 0.000 |
| 6) Total wrinkle area | 0.049 | 0.035 |
| 7) Total length of wrinkles | 0.003 | 0.027 |
| 8) Ra | 0.000 | 0.000 |
| 9) Rz | 0.001 | 0.000 |

3 A2. Nasolabial fold and forehead

| Parameter | *p*-value | |
| --- | --- | --- |
|  | 2 weeks | 4 weeks |
| 1) Average depth of wrinkles | 0.001 | 0.000 |
| 2) Mean depth biggest wrinkle | 0.002 | 0.000 |
| 3) Max. depth biggest wrinkle | 0.001 | 0.000 |
| 4) Total wrinkle count | 0.008 | 0.000 |
| 5) Total wrinkle volume | 0.012 | 0.002 |
| 6) Total wrinkle area | 0.018 | 0.029 |
| 7) Total length of wrinkles | 0.003 | 0.001 |
| 8) Ra | 0.002 | 0.000 |
| 9) Rz | 0.000 | 0.000 |

3 A3. Forehead

| Parameter | *p*-value | |
| --- | --- | --- |
|  | 2 weeks | 4 weeks |
| 1) Average depth of wrinkles | 0.004 | 0.000 |
| 2) Mean depth biggest wrinkle | 0.001 | 0.000 |
| 3) Max. depth biggest wrinkle | 0.008 | 0.000 |
| 4) Total wrinkle count | 0.002 | 0.000 |
| 5) Total wrinkle volume | 0.008 | 0.000 |
| 6) Total wrinkle area | 0.001 | 0.000 |
| 7) Total length of wrinkles | 0.002 | 0.003 |
| 8) Ra | 0.003 | 0.000 |
| 9) Rz | 0.002 | 0.000 |

3C. Skin sagging

| Time point | 2 weeks | 4 weeks |
| --- | --- | --- |
| *p*-value | 0.000 | 0.000 |

3D. Skin pigmentation

| Time point | 2 weeks | 4 weeks |
| --- | --- | --- |
| *p*-value | 0.000 | 0.000 |

3E. Dermal density

| Time point | 4 weeks |
| --- | --- |
| *p*-value | 0.000 |

3F. Mechanical imprint (pressure) relief

| Time point | 2 weeks | 4 weeks | 2 weeks vs. 4 weeks |
| --- | --- | --- | --- |
| *p*-value | 0.000 | 0.000 | 0.000 |

**For data in Figure 4. Analysis of parameters after 1 week of discontinuation of the product**

4A. Crow’s feet

| Parameter | *p*-value | | |
| --- | --- | --- | --- |
|  | 4 week | After 1 week of discontinuation of the product | 4 week vs. After 1 week of discontinuation of the product |
| 1) Ra | 0.048 | 0.045 | 0.620 |
| 2) Rmax | 0.003 | 0.001 | 0.087 |
| 3) Rz | 0.001 | 0.002 | 0.409 |
| 4) Rp | 0.000 | 0.002 | 0.296 |
| 5) Rv | 0.048 | 0.014 | 0.151 |

4C. Skin pigmentation

| Time point | 4 week | After 1 week of discontinuation of the product | 4 week vs. After 1 week of discontinuation of the product |
| --- | --- | --- | --- |
| *p*-value | 0.000 | 0.000 | 0.197 |

**For data in Figure 5. Analysis of parameters after application of the product once**

5A. Fine lines in the eye area

| Parameter | *p*-value |
| --- | --- |
|  | Immediately after application |
| 1) Ra | 0.000 |
| 2) Rmax | 0.000 |
| 3) Rz | 0.000 |
| 4) Rp | 0.000 |
| 5) Rv | 0.000 |

5C. Skin sagging

| Time point | Immediately after application |
| --- | --- |
| *p*-value | 0.000 |

5D. Skin gloss

| Time point | Immediately after application |
| --- | --- |
| *p*-value | 0.000 |

5E. Skin hydration

| Time point | Immediately after application |
| --- | --- |
| *p*-value | 0.000 |

**[2] Revision (Minor)**
The standard statistical term for average is mean.

**Response:** We agree with the reviewer’s contention that “mean” rather than “average” is the standard statistical term. Therefore, as suggested, we have replaced “average” with “mean” throughout the revised manuscript. (As shown below, we have marked the changes with yellow highlight.)

**(Line 192 on page 8 of the manuscript)**

**Materials and methods:**

**Clinical trial**

Twenty-one subjects (females, mean age, 50.95±5.54 years; age, 40–59 years) participated in the 4-weeks treatment test (two of twenty-three subjects dropped out [follow-up failure: #03 and #09]), and twenty subjects (mean age, 51.40±5.28 years; age, 40–59 years) participated in the discontinuation 1 week test after 4-weeks treatment (one more subject was dropped out [follow-up failure: #11]). Subjects who met the inclusion and exclusion criteria completed the study according to the protocol. The information and characteristics of the subjects were investigated using questionnaires.

However, for the skin wrinkle evaluation parameters, we have not replaced “average” with “mean” because in these cases the term is a part of the parameter name and, hence, a proper noun. The following are the names of the wrinkle parameters in the analysis program (The Primos software):

**(Line 267 on page 11 of the manuscript)**

1. Average depth of wrinkles

2. Mean depth biggest wrinkle

3. Max. depth biggest wrinkle

4. Total wrinkle count

5. Total wrinkle volume

6. Total wrinkle area

7. Total length of wrinkles

8. Ra (Arithmetic average)

9. Rz (Average maximum height of the profile)

**[3] Revision (Minor)**

Study procedure: State precisely the total number of subjects enrolled and analyzed instead of vaguely indicating “over 20 subjects.”

**Response:** Thank you for your comment. We would like to submit that we used “over 20 subjects” in accordance with the cosmetic clinical trial method guidelines followed in the Republic of Korea (Ministry of Food and Drug Safety MFDS, Guidelines for Cosmetics Human Application Test and Efficacy Test). The relevant text extracted from Ministry of Food and Drug Safety MFDS, Republic of Korea, Guidelines for Cosmetics Human Application Test and Efficacy Test is quoted below.

**(Part of contents in Ministry of Food and Drug Safety, MFDS)**

“For the number of subjects, valid data of 20 or more should be secured for statistical comparison.”

The number of people who participated in the actual test has been mentioned for the each evaluation item in the Results section. (The number of subjects participating in the trial is underlined.) The number of participating subjects for all evaluations was 21. However, in the evaluation of “after 1 week of discontinuation of product use,” one subject dropped out due to withdrawal of consent, and finally, 20 participants participated. We performed analysis on those 20 subjects.

**(Line 234 on page 10 of the manuscript)**

**Results**

**Analyses** **after treatment for 4 weeks**

**Analysis of wrinkles (Crow’s feet,** **nasolabial fold, and forehead)**

We conducted a test on 21 subjects to confirm the antiwrinkle effect of the product.

**Analysis of skin sagging**

We conducted a test on 21 subjects to confirm the skin-lifting effect of the product.

**Analysis of skin pigmentation**

We investigated the effect of the product in improving skin pigmentation in 21 subjects.

**Analysis of dermal density**

We conducted a study on 21 subjects to evaluate the effect of the product in improving dermal density.

**Analysis of mechanical imprint (pressure) relief**

We conducted a test on 21 subjects to evaluate the effect of the product on the mechanical imprint (pressure) relief.

**Analysis after 1 week of discontinuation of the product**

**Analysis of Crow’s feet**

We conducted a study on 20 subjects to evaluate the improvement in Crow’s feet.

**Analysis of skin pigmentation**

We conducted a study on 20 subjects to evaluate the improvement in pigmentation..

**Analysis of the effects after single application of the product**

**Analysis of fine lines in the eye area**

We confirmed the effect of the product use in improving the fine wrinkles in 21 subjects.

**Analysis of skin sagging**

We checked the facial lifting effects of the product in 21 subjects.

**Analysis of skin gloss**

We evaluated the skin radiance improvement effect of the product in 21 subjects.

**Analysis of skin surface to a depth of 2.5 mm hydration**

We evaluated the effect of the product in improving the skin surface to a depth of 2.5 mm hydration in 21 subjects.

**[4] Revision (Minor)**
Safety Evaluation: Provide an interpretation for “Range of response = 0.00.”

**Response:** Safety evaluation aimed at evaluating the primary skin irritation potential of the test materials using a primary patch test for the human skin. We performed a 24-hours occlusive patch test. After removing the patch, each test region was observed after 30 minutes and 24 hours, according to the PCPC guidelines. Over 30 subjects were selected according to the recommendations in the MFDS guidelines. We derived the results with reference to Tables 1 and 2 below. Therefore, using this test procedure for the primary irritation potential for human skin, the test product was found to be in the “none to slight” range (range of response = 0.00). We have added this information the Results section (Line 353 on page 15 of the manuscript) and attached Supporting Text file.

**(Contents in Supporting Text 1 and Supporting Table 2.)**

**STUDY PROCEDURE:**

1. The test region (back skin) was washed with 70% ethanol and dried.

2. “Olivapep-Wr” (20 µL) was distributed in IQ Ultra^TM^ Patch Test Units (Chemotechnique Diagnostics AB, Sweden).

3. The IQ chamber was attached to the test site for 24 hours.

4. After removing the patch and marking the test region with a skin marker (skin marker pen, DeRoyeal, USA), each test region was assessed under a magnifying lens (BD -FS3, ARO, Korea) at 30 min and 24 h after the removal of the patch.

Skin irritation potential was evaluated according to the PCPC guidelines (Table 1).

| Irritation Score  (IS) | = | ∑ (Score × No. of responders) | × 100 | |
| --- | --- | --- | --- | --- |
|  |  | 4 (Maximum score) × N (Total No. of subjects) |  |  |
| Mean of Irritation Score  (MIS) | = | (Irritation score after 30 minutes of patch removal  + Irritation score after 24 hours of patch removal) | |  |
|  |  | 2 (Time point) | |  |

The irritation score was derived using the formula given above, and the result was inferred based on the criteria listed in Table 2.

Table 1. Skin Irritation Scoring System

| Score | Skin Irritation |
| --- | --- |
| 0 | No sign of inflammation; normal skin |
| 0.5 | Glazed appearance of the sites, or barely perceptible erythema |
| 1 | Slight erythema |
| 2 | Moderate erythema, possibly with barely perceptible edema at the margin, papules may be present |
| 3 | Moderate erythema, with generalized edema |
| 4 | Severe erythema with severe edema, with or without vesicles |
| 5 | Severe reaction spread beyond the area of the patch |

Table 2. Determination Criteria for Skin Primary Irritation Potential

| Range of Irritation | Determination |
| --- | --- |
| 0.00 ≤ MIS < 0.87 | None to slight |
| 0.87 ≤ MIS < 2.42 | Mild |
| 2.42 ≤ MIS < 3.44 | Moderate |
| 3.44 ≤ MIS | Severe |

*Ref.: International Journal of Cosmetic Science 2014, 36(1), 62-67 ^8)^*

Using this test procedure for the primary irritation potential for human skin, the test product was found to be in the “none to slight” range (range of response = 0.00).

**[5] Revision (Minor)**
State and justify the study’s target sample size with a pre-study statistical power calculation. The power calculation should include: sample size, alpha level (indicating one or two-sided), minimal detectable difference and statistical testing method.

**Response:** We apologize for insufficient application of statistical techniques for justifying the target sample size and for not introducing our study methodology in detail. We have added information to reinforce our main results (Line 142 on page 6 of the manuscript). We hope that the changes would address the reviewer’s concern.

**(Line 142 on page 6 of the manuscript)**

**Sample size calculation**

Although there has been no study on the antiaging potential of TEENIALL (ascorbic acid and collagen), prior to conducting clinical research, we calculated the sample size based on our *in vivo* study on the relationship between collagen synthesis and TEENIALL (22). We originally calculated that for each group to have a 95% power and 0.05 alpha to report a 44.5%–83.1% (18.7% in certain experiments) improvement in collagen synthesis (229.1 [SD 31.5] ng/mL for control group versus 269 ng/mL in certain experiments for treatment group), at a 5% significance level, we would need to enroll 16 patients in each group. Considering the various exclusion conditions (30%), finally, we included 21 participants.

**[6] Revision (Minor)**

To assist in the review process, add line numbering to the document.

**Response:** We would like to thank the reviewer for the comments. We have added line numbers to the Word document.

**<Reviewer 2>**

**[7] Revision (Minor)**

The source of the cell lines are not given.

**Response:** We would like to thank the reviewer for the comments. We have added the source of the cell lines in the revised manuscript.

**(Line 129 on page 6 of the manuscript)**

***In vitro* and *ex vivo* analysis**

**Measurement of collagen synthesis**

Human normal fibroblasts (neonatal; Gibco, C0045C) were treated with DMEM containing TEENIALL at a concentration of 0.01, 0.02, or 0.05%. Collagen synthesis was measured using an ELISA kit (Procollagen Type I C-peptide [PIP] EIA Kit, TaKaRa, Tokyo, Japan).

**[8] Revision (Minor)**

Use of generic names for the evaluated products/ details of components could make it of better use for the scientific community to advance science.

**Response:** We would like to thank the reviewer for the pertinent suggestion. We have used generic names instead of “TEENIALL” and “Naticol^®^.” Moreover, we have added a description about the ascorbic acid and collagen mixed component, which we refer to as TEENIALL, in the revised manuscript.

**(Line 50 on page 2 of the manuscript)**

**Abstract:**

Based on the results of *in vitro* and *ex vivo* experiments and clinical trials, we show that the product containing ascorbic acid and collagen was effective in alleviating skin aging.

**(Line 113 on page 5 of the manuscript)**

**Materials and methods**

**Test product**

TEENIALL (TEENIALL Double Concentrate Vitamin C Youth Ampoule, WELBORN, Korea) is composed of powder type ascorbic acid and liquid type collagen (Naticol BPMG). Ascorbic acid contained in this product is in the form of fine particles, with a diameter of 37 µm, and the molecular weight of collagen is 1.74 Mw/Mn. In clinical trial, this product was divided into powder and liquid components, so that both formulations could be mixed and applied immediately before use.

**(Line 234 on page 10 of the manuscript)**

**RESULTS:**

***In vitro* and *ex vivo* analyses**

**Cell penetration assay**

...indicating the penetration of the product (TEENIALL) into these cells (Fig 2B, S1 Table).

**(Line 256 on page 11 of the manuscript)**

**Fig 2.** (A) Collagen and ascorbic acid have beneficial effect on skin aging.

**(Line 414 on page 17 of the manuscript)**

**Conclusions**

We believe that there will be better results if the improvement effect is measured when collagen and ascorbic acid are used over a long time.

**<Reviewer 3>**

**[9] Revision (Minor)**

In the introduction, page 2, the sentence starting with “Ascorbic acid is a representative….” can be given as a separate paragraph.

**Response:** We appreciate this suggestion. We have presented the text mentioned by the reviewer in a separate paragraph the Introduction section (Line 80 on page 4 of the manuscript).

**(Line 80 on page 4 of the manuscript)**

**Introduction**

In particular, Naticol^®^ BPMG is composed of collagen type 1 and type 3, which are the main components of the dermis. It is produced from high quality raw materials (fish skin) and through specific enzymatic hydrolysis (15). ddddddddddddddddddddddddddddddddddddd

Ascorbic acid is a representative water-soluble antioxidant. Two different mechanisms are described for the antioxidant properties of ascorbic acid (16).

**[10] Revision (Minor)**

The products Naticol ® and Quali®-C, are mentioned as “raw materials” in most of the places. Please change that to “products” or any other word of similar meaning.

**Response:** We would like to thank the reviewer for the comment. We have changed “raw materials” to specific terms (“Naticol^®^ and ascorbic acid”).

**(Line 98 on page 4 of the manuscript)**

**Introduction**

First, experiments were conducted using cells and tissues to confirm the permeability and collagen synthesis ability of Naticol^®^ and ascorbic acid, and thereafter, clinical studies were conducted in human subjects.

We evaluated the synergistic effect of Naticol^®^ and ascorbic acid and the benefits of using the special container on the antiaging effect. We also assessed whether the effect was maintained even after the discontinuation of the product.

**[11] Revision (Minor)**

In the study procedure the sentences” In this study, treatment was applied once, 2 and 4 weeks, the study was conducted as a single test. Over 20 subjects meeting the inclusion and exclusion criteria were enrolled” and “For 4 weeks, the product was applied to subjects on a daily basis around the whole face after washing the face twice a day” are unclear and misleading.

**Response:** We apologize for the lack to clarity. We have revised the description and hope that it conveys the intended meaning.

**(Line 156 on page 7 of the manuscript)**

**Clinical trials**

**Study procedure**

Over 20 subjects meeting the inclusion and exclusion criteria were enrolled. For 4 weeks, the product was applied to subjects on a daily basis around the whole face after washing the face twice a day. In this study, treatment was applied for 4 weeks; the study was conducted as a single test and the measurements were made immediately after the application of the product once, and 2 and 4 weeks later.

**[12] Revision (Minor)**

Please correct the grammatical errors in the manuscript. There are several grammatical errors found. The English language is generally unsatisfactory. I suggest rewriting the conclusion. The line starting with “In summary….can be corrected”

**Response:** We acknowledge that there were language issues in the manuscript. In accordance with the reviewer’s comment, we have employed a professional scientific editing service to ensure that the language of the revised manuscript meets the publishable standards.

**(Line 406 on page 17 of the manuscript)**

**Conclusions**

The combined use of ascorbic acid and collagen resulted in skin whitening and wrinkle improvement, as well as in the improvement of skin elasticity, radiance, and moisturizing effects. In addition, the effects of ascorbic acid and collagen were synergistic and were maintained even after discontinuation of the product. Although the effects of the combination of collagen and ascorbic acid were clinically confirmed, the molecular mechanisms underlying the effects of each were not confirmed. Therefore, it would be good to check the effects of each component separately to elucidate the exact mechanism. Moreover, it may be necessary to conduct a study comparing the data for each. We believe that there will be better results if the improvement effect is measured when collagen and ascorbic acid are used over a long time.
